# Amiodarone-Associated Optic Neuropathy in a Patient With Associated Arrhythmia

**DOI:** 10.7759/cureus.55819

**Published:** 2024-03-08

**Authors:** Sharvil Patel, Riaz Mahmood

**Affiliations:** 1 Internal Medicine, Northeast Georgia Medical Center Gainesville, Gainesville, USA

**Keywords:** drug-induced optic neuropathy, sudden vision loss, side effects of amiodarone, acute vision loss, amiodarone associated optic neuropathy, neurological side effects of amiodarone, toxic optic neuropathy

## Abstract

Amiodarone is a commonly used antiarrhythmic used to treat atrial fibrillation and ventricular tachycardias. While this agent can present with pulmonary, thyroid, and hepatic side effects, it can also, less commonly cause neurologic toxicity, particularly optic neuropathy. Optic neuropathy can manifest as acute vision loss. The management of amiodarone-associated optic neuropathy (AAON) includes early recognition of symptom manifestation so that the medication can be discontinued promptly. Here, we describe a case of a 64-year-old male who developed acute onset complete left-sided vision loss after initiation of amiodarone.

## Introduction

Amiodarone is commonly used for arrhythmias but has a toxic side effect profile. Long-term oral therapy can lead to accumulation in tissues, leading to thyroid dysfunction, peripheral neuropathy, pulmonary toxicity, ataxia, photosensitivity, and gastrointestinal problems [1]. In rare circumstances, it can cause ocular toxicity, which most commonly presents as corneal deposits called whorl keratopathy or corneal verticillata [2]. Other ocular side effects include corneal microdeposits, subcapsular opacities, dysthyroid eye disease, enlargement of extraocular muscles, and multiple chalazia [3]. Of greater concern is amiodarone-associated optic neuropathy (AAON), which is the most threatening ocular side effect [4]. AAON typically results in monocular or bilateral decreased visual acuity occurring acutely or insidiously [4]. AAON remains a controversial and difficult diagnosis due to the variability in presentation [5]. We present a case of a 64-year-old male with a history of ventricular tachycardia on amiodarone who presented with acute onset left-sided vision loss.

## Case presentation

**Figure 1:**
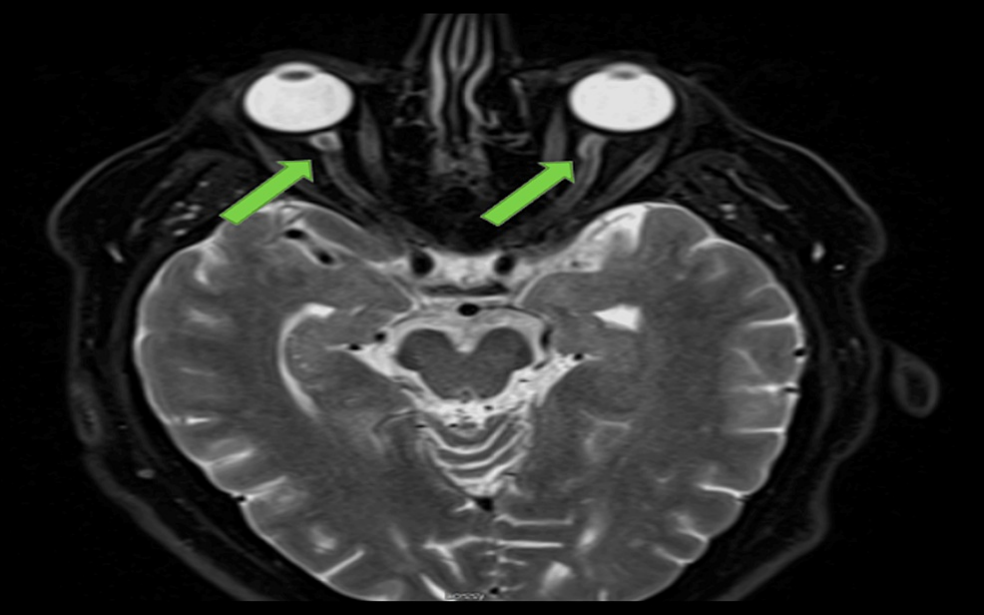
MRI brain/orbit showing minimal to mild wavy contour noted to the optic nerve with tram track appearance, green arrows on T2-weighted imaging suggestive of mild optic nerve edema, left greater than right

A 64-year-old male with a past medical history significant for ventricular tachycardia, diabetes, hypertension, non-ST-elevation myocardial infarction s/p stent placement to the right coronary artery, and chronic kidney disease presented to the emergency department (ED) for the gradual onset of complete vision loss in the left eye over four days. Associated symptoms included right-sided temporal headache and photophobia. An ophthalmologist evaluated the patient earlier that day and referred him to the ED for further evaluation. In the ED, his vitals were notable for blood pressure of 210/112 mmHg, heart rate of 60 bpm, and respiratory rate of 22 breaths per minute. He reported being compliant with his antihypertensives, which included hydralazine and metoprolol. On physical examination, pupils were 6 mm bilaterally, sluggish on the right, and positive swinging flashlight test in the left eye. A fundoscopic exam revealed prominent vessels with possible retinal hemorrhage in the left eye. Laboratory values on presentation are depicted in Table 1. The workup revealed an elevated creatinine of 1.92 mg/dL. The sedimentation rate was elevated at 26 mm, and C-reactive protein was within the normal range. He was started on IV methylprednisolone every six hours. MRI of the brain orbit with and without contrast revealed enhancement of possible mild edema of the left optic nerve and minimal to mild wavy contour noted to the optic nerve with a tram-track appearance suggestive of mild optic nerve edema, left greater than right (Figure 1). The patient was admitted for a hypertensive emergency. Neurology and ophthalmology were consulted. He underwent a left temporal artery biopsy, paraneoplastic antibody evaluation, meningitis polymerase chain reaction panel, and lumbar puncture, which were all negative. There was high suspicion of AAON. He had been on amiodarone for four months. Cardiology was consulted. His amiodarone was discontinued, and his metoprolol was increased. He was discharged home with an event monitor. At his follow-up appointment, the patient reported mild improvement in vision.

**Table 1:** Table demonstrating significant laboratory values upon initial presentation BUN: Blood urea nitrogen; ALT: Alanine transaminase; AST: Aspartate transaminase; CRP: C-reactive protein; ESR: Erythrocyte sedimentation rate

Laboratory tests	Value	Reference range
Creatinine	1.92 mg/dL	0.80-1.30 mg/dL
BUN	26.0 mg/dL	3.0-23.0 mg/dL
Potassium	4.0 mmol/L	3.5-5.2 mmol/L
ALT	146 U/L	13-61 U/L
AST	92 U/L	0-48 U/L
CRP	<0.40 mg/dL	0.00-0.60 mg/dL
ESR	26 mm	0-20 mm

## Discussion

Amiodarone is one of the most widely used antiarrhythmics used to treat atrial fibrillation and ventricular tachycardias [6]. It is believed that the amphophilic properties of the drug cause it to be widely distributed throughout the body's tissues, including the eye [6]. Amiodarone induces phospholipidosis, resulting in intracellular accumulation of phospholipids with lamellar bodies [7]. The organs affected by phospholipidosis exhibit inflammatory reactions and histopathological changes [7]. Histopathology has identified multiple lamellated inclusion bodies in large axons of the optic nerve in patients taking amiodarone, while demyelination or loss of large axons was not seen [8]. This finding highlights that amiodarone may have a neurotoxic effect on the optic nerve via drug-induced lipidosis.

AAON is characterized by an insidious onset of vision loss with slow progression and optic disc swelling. Usually, this condition initially presents unilaterally and can eventually result in simultaneous vision loss in both eyes. While many cases result in mild optic nerve dysfunction, others have experienced permanent vision loss. One study noted that the median interval for onset of optic neuropathy was approximately four months after initiating amiodarone [9]. Studies have reported the incidence of amiodarone optic neuropathy as high as 2.0%; however, the exact incidence is unknown [10].

The diagnosis of AAON is clinical, and the variability in presentation makes it a controversial diagnosis. An observational case series of 55 cases noted that 40% of the patients presented with sudden vision loss, 80% of the patients had optic disc edema, and 5 patients presented with elevated intracranial pressure on lumbar puncture [9]. A wide spectrum of optic nerve involvement has been reported in patients with AAON, including asymptomatic optic disc edema, unilateral optic neuropathy, and bilateral simultaneous or sequential optic neuropathy [6]. In addition, AAON requires distinction from non-arteritic ischemic optic neuropathy (NAION), which is the most common cause of optic neuropathy in individuals over the age of 50 [11]. AAON differs from NAION in that AAON occurs more often in men and with systemic hypertension, while NAION has equal sex predilection [4]. In addition, optic disc swelling may persist for 1-8 months in AAON, with a median duration of three months, whereas disc swelling in NAION usually resolves within 2-6 weeks [2]. Patients with NAION also tend to have small, crowded optic discs [2].

AAON is classified into five clinical categories, classified by either temporal characteristics or optic nerve appearance. The most common form of AAON is insidious onset, in which these patients commonly have bilateral and simultaneous optic disc edema [9]. The second most common form is an NAION-like picture, which presents as an acute unilateral or bilateral visual loss [9]. The third type of AAON is retrobulbar optic neuropathy, which is the most difficult to diagnose and requires neuroradiologic imaging as well as blood studies to rule out other etiologies of visual field loss in the setting of normal optic nerves [9]. The fourth category is classified as increased intracranial pressure greater than 200 mmH_2_O and the fifth category is delayed progressive onset, in which these patients can develop optic disc edema several days to weeks after amiodarone is withdrawn [9]. Annual ophthalmology screening for patients should be considered for patients who are taking amiodarone. If the diagnosis is suspected, cardiology consultation regarding the feasibility of discontinuing amiodarone with alternative medication or treatment should be explored.

## Conclusions

Although rare, this case illustrates the potential for optic neuropathy with amiodarone and the value of being vigilant to its toxic side effect profile. Additionally, because amiodarone is a widely used drug for life-threatening arrhythmias, risk stratification should be considered in determining discontinuation of the drug, decreasing the dosage, or using alternate anti-arrhythmic modalities when appropriate. It is imperative to seek prompt consultation with a cardiologist when AAON is suspected.

## References

[1] Nagra PK, Foroozan R, Savino PJ, Castillo I, Sergott RC (2003). Amiodarone induced optic neuropathy. Br J Ophthalmol.

[2] Wang AG, Cheng HC (2017). Amiodarone-associated optic neuropathy: clinical review. Neuroophthalmology.

[3] Passman RS, Bennett CL, Purpura JM (2012). Amiodarone-associated optic neuropathy: a critical review. Am J Med.

[4] Wang Wang, Rui Rui (2024). Amiodarone associated optic neuropathy. EyeWiki.

[5] Eryılmaz T, Atilla H, Batıoǧlu F, Günalp I (2000). Amiodarone-related optic neuropathy. Jpn J Ophthalmol.

[6] Murphy MA, Murphy JF (2005). Amiodarone and optic neuropathy: the heart of the matter. J Neuroophthalmol.

[7] Anderson N Anderson N,  Borlak J  Borlak J Drug-induced phospholipidosis. FEBS Letters.

[8] Mansour AM, Puklin JE, O'Grady R (1988). Optic nerve ultrastructure following amiodarone therapy. J Clin Neuro-Ophthal.

[9] Johnson LN, Krohel GB, Thomas ER (2004). The clinical spectrum of amiodarone-associated optic neuropathy. J Natl Med Assoc.

[10] Mindel JS, Anderson J, Hellkamp A (2007). Absence of bilateral vision loss from amiodarone: a randomized trial. Am Heart J.

[11] Biousse V, Newman NJ (2015). Ischemic optic neuropathies. N Engl J Med.

